# Climatic Warming-Induced Drought Stress Has Resulted in the Transition of Tree Growth Sensitivity from Temperature to Precipitation in the Loess Plateau of China

**DOI:** 10.3390/biology12101275

**Published:** 2023-09-25

**Authors:** Qindi Zhang, Shaomin Fu, Hui Guo, Shaoteng Chen, Zongshan Li

**Affiliations:** 1 College of Life Sciences, Shanxi Normal University, Taiyuan 030031, China; nyzqd@126.com (Q.Z.); fuuu1999@163.com (S.F.); ghfsmzlh@163.com (H.G.); 2 State Key Laboratory of Urban and Regional Ecology, Research Center for Eco-Environmental Science, Chinese Academy of Sciences, Beijing 100085, China; chtcst@163.com; 3 Institute of International Rivers and Eco-Security, Yunnan University, Kunming 650500, China; 4 National Observation and Research Station of Earth Critical Zone on the Loess Plateau in Shaanxi, Xi’an 710061, China

**Keywords:** climate change, tree radial growth, Loess Plateau, warming phases

## Abstract

**Simple Summary:**

Although two warming phases have been confirmed over the past century, the responses of tree growth in drylands remain uncertain. To investigate this, we utilized the tree-ring chronology in the Loess Plateau of China to explore the changes in tree growth–climate relationships during these warming phases. Our findings indicate that tree growth rates primarily increased during the first warming phase (1910–1940) and decreased during the second phase (1970–2000). Furthermore, we observed that temperature was the main influencing factor for tree growth during the first phase, whereas drought played a more significant role during the second phase. These temporal changes emphasize the importance of water availability for tree growth in drylands and suggest that reductions in precipitation will further exacerbate the adverse effects of climate warming on tree growth.

**Abstract:**

Ongoing climate warming poses significant threats to forest ecosystems, particularly in drylands. Here, we assess the intricate responses of tree growth to climate change across two warming phases (1910–1940 and 1970–2000) of the 20th century in the Loess Plateau of China. To achieve this, we analyzed a dataset encompassing 53 ring-width chronologies extracted from 13 diverse tree species, enabling us to discern and characterize the prevailing trends in tree growth over these warming phases. The difference in the primary contributors over two warming phases was compared to investigate the association of tree growth with climatic drivers. We found that the first warming phase exerted a stimulating effect on tree growth, with climate warming correlating to heightened growth rates. However, a contrasting pattern emerged in the second phase as accelerated drought conditions emerged as a predominant limiting factor, dampening tree growth rates. The response of tree growth to climate changed markedly during the two warming phases. Initially, temperature assumed a dominant role in driving the tree growth of growth season during the first warming phase. Instead, precipitation and drought stress became the main factors affecting tree growth in the second phase. This drought stress manifested predominantly during the early and late growing seasons. Our findings confirm the discernible transition of warming-induced tree growth in water-limited regions and highlight the vulnerability of dryland forests to the escalating dual challenges of heightened warming and drying. If the warming trend continues unabated in the Loess Plateau, further deterioration in tree growth and heightened mortality rates are foreseeable outcomes. Some adaptive forest managements should be encouraged to sustain the integrity and resilience of these vital ecosystems in the Loess Plateau and similar regions.

## 1. Introduction

The Fifth Assessment Report of the Intergovernmental Panel on Climate Change (IPCC AR5) states that the world experienced two distinct warming phases during the 20th century [1]. The early part of the 20th century (1910–1940) exhibited a clear warming trend in global temperatures (the first warming phases hereafter). In the second half of the 20th century (1970–2000), global temperatures once again rose significantly (the second warming phase hereafter). The impact of climate change, notably in drylands, reverberates acutely across forest ecosystems due to the inherent intricacies of tree longevity, which constrains swift adaptation to energy and water constraints [2,3]. Climate warming impacts the health and functioning of dryland forest ecosystems with consequences on substantial ecosystem services for human well-being, including water and soil conservation as well as carbon sequestration and climate change mitigation [4,5,6]. Climate projections suggest that increases in temperature across the boreal forest region are likely to exceed 2 °C by the end of the century [7]. Climate warming in the future will bring great uncertainty to the forest dynamics. Therefore, understanding the potential effects of climate warming on forest ecosystems is a long-standing goal of the forest ecological research agenda in water-limited regions.

Rising temperatures have multifaceted effects on plants, including increased energy inputs, an elevated vapor-pressure deficit, and decreased soil moisture [8]. Tree radial growth has strong connections to climate change. In this sense, tree ring indices provide higher-resolution information on tree growth response to changing climate [9]. Despite numerous dendroclimatological studies aimed at elucidating tree growth-climate relationships in water-limited regions, there persists considerable variability in forest responses to climate warming [10,11,12]. In general, elevated temperatures tend to foster growth in cool regions, such as the Tibetan Plateau [13,14,15]. However, it is important to note that beyond a certain temperature threshold, tree growth is adversely affected, primarily due to diminishing net photosynthetic gains [12]. Furthermore, as energy inputs increase, the influence of water availability on plant growth often surpasses that of the temperature [3]. Consequently, higher temperatures are generally associated with reduced tree growth in water-limited regions, culminating in diminished growth rates and an accelerated incidence of tree mortality [16,17,18]. Nevertheless, the confluence of climate warming also introduces a layer of uncertainty, encompassing shifts in precipitation patterns, storm intensity, drought frequency, and the severity of extreme events. The intricacies of plant growth are attributed to complex interactions between energy and water availability rather than a singular driver [19]. In the context of global warming, although it is appreciated that temperature always interacts with other factors in producing an effect, large uncertainties still remain around the relative importance of the climatic drivers of plant growth over time. This ambiguity is particularly pronounced in water-limited regions such as the Loess Plateau.

The Loess Plateau, situated in the arid and semi-arid region of Northwestern China, holds immense significance for soil-erosion control and vegetation restoration, playing a pivotal role in ecological security and development [20,21]. Over the past few decades, temperatures in this region have experienced a substantial increase, with a warming rate exceeding twice the Northern Hemisphere average [22]. Climate projections for the Loess Plateau augur a continued rise in temperatures throughout the plateau, with the northern and eastern regions prognosticated to undergo the most pronounced changes [23]. Against this backdrop, the objectives of the present study were to investigate how tree growth responds to climate variability across two warming phases. To achieve this, we amassed a dataset comprising 53 ring-width chronologies derived from 13 tree species native to the Loess Plateau, encompassing the two warming phases prevalent during the 20th century. Drawing from previous research, we formulated two hypotheses: (1) Climate warming has incurred a decline in tree growth, and (2) The climate drivers governing tree growth exhibit disparities between these two warming phases. To test these hypotheses, we initially scrutinized the trends in climate parameters and tree growth across the two warming phases. Subsequently, we delved into the intricacies of the tree growth–climate relationships, discerning any alterations that transpired between the two phases. 

## 2. Materials and Methods

### 2.1. Study Area

**Figure 1:**
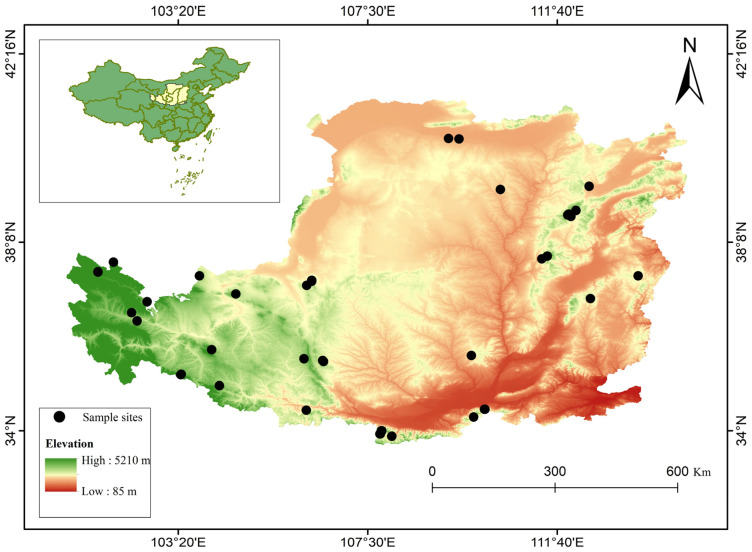
Distribution of sample sites of the Loess Plateau of China.

This study was conducted in the Loess Plateau of China (33.72°–41.27° N and 100.90°–114.55° E) (Figure 1). The average altitude is around 1000 m, decreasing from northwest to southeast. The Loess Plateau has a typical warm temperate continental monsoon climate. The mean annual temperature (MAT) exhibits regional variability, ranging from 3.6 °C in the northwest to 14.3 °C in the southeast. Mean annual precipitation (MAP) ranges from 123.3 mm in the northwest to 948.9 mm in the southeast [24]. More than 60% of precipitation falls between July and September. The vegetation distribution within this region displays a clear zonal pattern, with a diverse array of tree genera like *Pinus*, *Abies*, *Picea*, and *Larix* widely scattered throughout the forested areas owing to the intricate interplay between topography and climatic factors [25].

### 2.2. Chronological Data

The tree-ring width chronologies utilized in this study were exclusively derived from the Loess Plateau (Figure 1, Table S1). Initially, the tree-ring width chronologies specific to this region were sourced from the International Tree-ring Data Bank (ITRDB). Additionally, relevant studies pertaining to tree-ring analysis in the Loess Plateau were scanned and retrieved from Web of Science and the China National Knowledge Infrastructure (https://www.cnki.net/, accessed on 23 September 2022). These tree-ring data were subsequently digitized from graphical representations of the tree-ring chronologies using GetData software (v.2.26; https://soft.3dmgame.com/down/167665.html, accessed on 14 June 2022).

To ensure the integrity of the dataset for subsequent analyses, only tree-ring width chronologies adhering to the following criteria were retained: (a) Chronologies exhibiting total ring-width and (b) Chronologies encompassing both warming periods 1910–1940 and 1970–2000. After screening, 95 chronological series were preserved. Subsequently, 53 tree-ring width chronosequences with positive growth rates during the first warming phase were selected from the 95 sequences. Eventually, a total of 53 chronology series were deemed suitable for inclusion. These selected chronological series represent 13 species belonging to 7 genera. In order to compensate for inter-species variations and account for age-related growth patterns, all chronology series underwent normalization and standardization procedures.

### 2.3. Climate Data

We quantified the response of tree growth to three climate parameters: temperature, precipitation, and standardized precipitation evapotranspiration index (SPEI). The SPEI is an index that can be used as a measure of changing climate characteristics to describe long-term meteorological evapotranspiration increase and decrease trends. It can be used to measure changes in drought or flooding in an area to judge future changes in the area’s water resources, with smaller SPEI values indicating a drier climate. These parameters were used as metrics of energy and water availability, and drought, respectively. The monthly temperature, precipitation, and SPEI data for each sampling site were obtained from the Climatic Research Unit (CRU) time series (TS) version 4.05, available at a 0.5 × 0.5° grid size. The data cover the period from 1900 to 2020 and can be accessed through the website https://www.uea.ac.uk/web/groups-and-centres/climatic-research-unit/data (accessed on 22 September 2023). To facilitate our analysis, we curated a 17-month dataset encompassing the period from the prior June to the current October. Within this dataset, we derived a 5-month sliding window, computing the average climate data.

### 2.4. Statistical Analysis

In this study, our primary objective is to comprehensively evaluate the disparities in tree radial growth characteristics and climate sensitivity within the Loess Plateau during two warming phases. To achieve this, we employed a range of statistical analyses. Firstly, linear regression was employed to discern the temporal trends of three climate factors: temperature, precipitation, and SPEI, as well as tree growth during the two warming periods. Then, Pearson correlation analysis was conducted to provide insights into the relationship between tree responses and climate sensitivity. Finally, paired-sample *t*-tests were utilized to explore the variability in the correlations of tree-ring index with temperature, precipitation, and SPEI across the months in the two warming phases. All of the above statistical tests were conducted using SPSS version 25.0 (IBM Corporation, New York, NY, USA).

## 3. Results

### 3.1. Trends in Climate and Tree Growth

**Figure 2:**
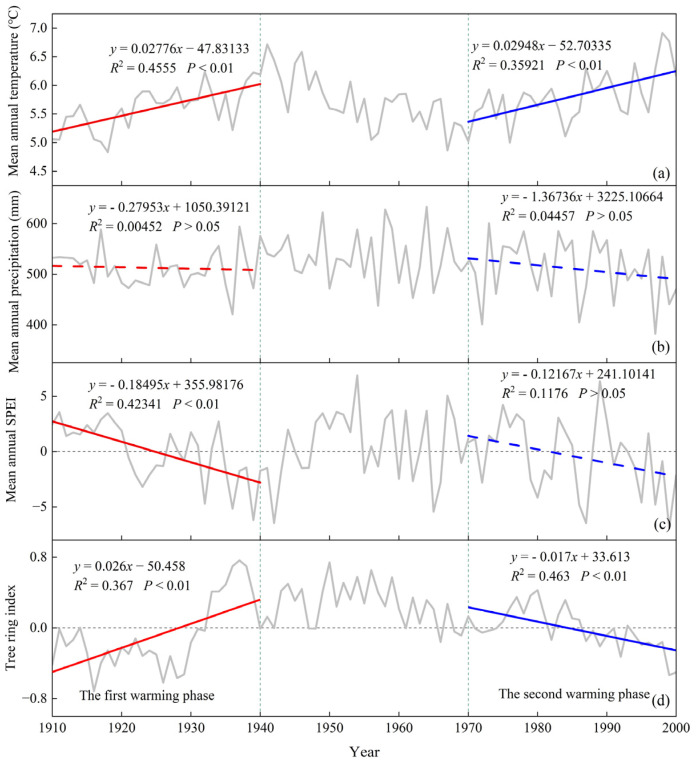
Climate change and tree growth trends in two warming phases. (**a**) Trends in the MAT; (**b**) Trends in the MAP; (**c**) Trends in the SPEI; (**d**)Trends in tree growth. The red trend line represents the fitted line of climate and tree growth rates for the first warming phase, while the blue colour similarly represents the second warming phase.

In Figure 2, we present trends in climate and tree growth observed during two distinct warming phases in the Loess Plateau. MAT continued to rise steadily in both phases, from a value of 5.607 ± 0.206 °C in the first warming phase to 5.807 ± 0.232 °C in the second warming phase. MAP did not change significantly with time during the first warming phase, while it showed an insignificant decreasing trend during the second warming phase. SPEI showed a decreasing trend in both warming phases. Specifically, MAP decreased from 512.295 ± 0.226 mm to 510.902 ± 0.225 mm, while SPEI decreased from −0.039 ± 0.224 to −0.409 ± 0.240. Tree growth rates exhibited divergent patterns during these warming phases (Figure 2d). In the first warming phase, there was a notable upward trend in overall tree growth rates (*p* < 0.01). However, in the second phase, tree growth rates demonstrated a significant downward trend (*p* < 0.01). The average tree growth rate increased from 0.027 ± 0.020 in the first warming phase to −0.016 ± 0.032 in the second phase (Table S2). With regards to individual tree species, the majority displayed an initial increase in growth rates followed by a subsequent decrease, with the exception of *Abies chensiensis* and *Abies fargesii* (Figure S1).

### 3.2. Spatial Pattern of Growth Rate and Climatic Sensitivity

**Figure 3:**
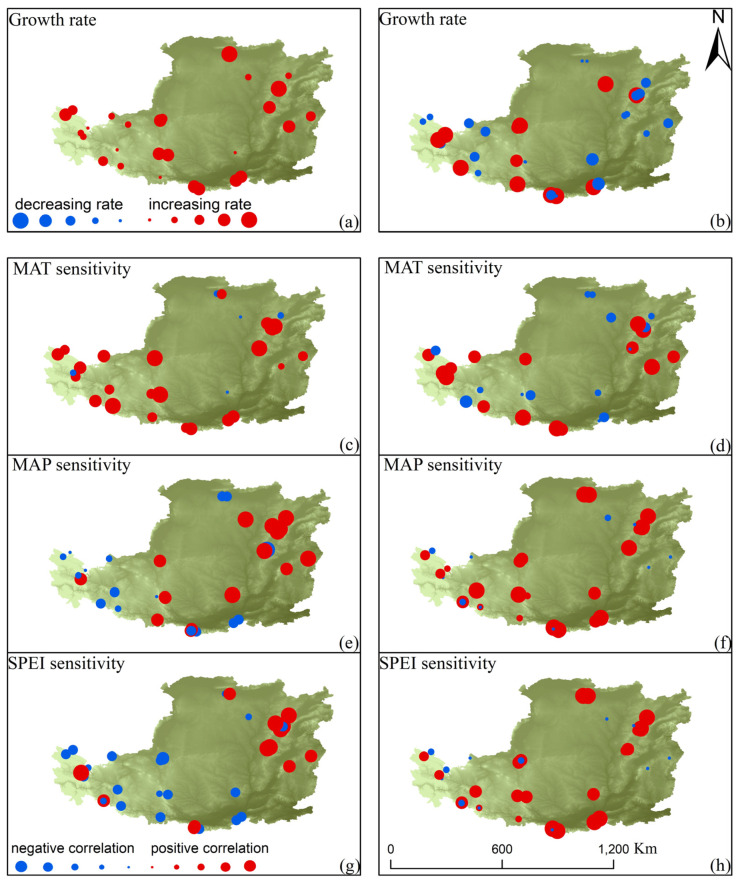
Spatial patterns of tree growth rates and climate sensitivity in two climate warming phases. MAT: temperature; MAP: precipitation. In graphs (**a**,**b**), the red and blue dots represent positive and negative tree growth rates, respectively, and the magnitude implies the size of the absolute value of the growth rate. In the (**c**–**h**) plot, the red and blue points represent the positive or negative response of tree growth sensitivity to climatic factors, respectively, and the magnitude implies the size of the absolute value of climate sensitivity.

Many of the tree ring sites that exhibited positive growth rates in the first warming phase transitioned to negative growth rates in the second phase (Figure 3a,b). The correlation between tree growth rates and MAT showed a spatially positive relationship in the first warming phase, which weakened significantly in the second phase (Figure 3c,d). Conversely, the response of tree growth rates to water availability (MAP and SPEI) considerably improved in the second warming phase (Figure 3f,h). Overall, the sensitivity of tree growth to climate shifted from temperature in the first warming phase to precipitation and SPEI in the second phase.

### 3.3. Relationships between Tree Growth and Climate

**Figure 4:**
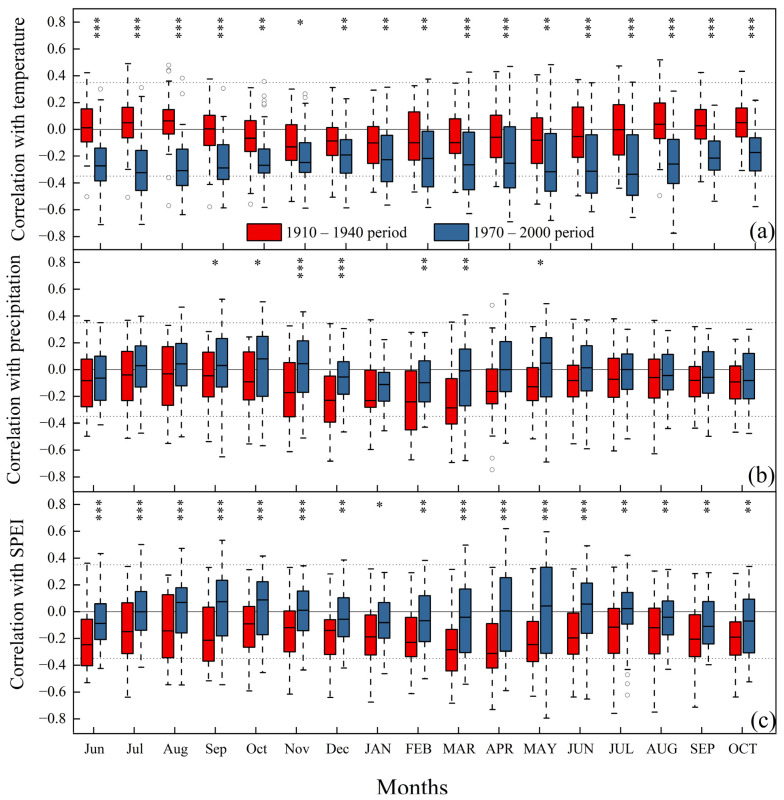
Correlation between tree rings index and climate in two warming phases. (**a**) Rings width and temperature correlation; (**b**) Rings width and precipitation correlation; (**c**) Rings width and SPEI correlation. Different asterisks indicate differences in climate sensitivity between the two warming phases, * *p* < 0.05, ** *p*< 0.01, and *** *p* < 0.001. Lowercase abbreviated months represent the previous year, while uppercase abbreviated months represent the current year. The circles indicate outliers.

The pattern of the correlations differed significantly between the two warming phases (Figure 4). During the first warming phase, tree growth had a correlation of 0.082 ± 0.206 with temperature, which changed to −0.131 ± 0.232 in the second warming phase. Additionally, the relationship between trees and both precipitation and SPEI shifted from negative (−0.021 ± 0.226 and −0.085 ± 0.224, respectively) to positive (0.073 ± 0.225 and 0.077 ± 0.240, respectively) correlations (Table S2). The tree ring index in the first warming phase exhibited a positive correlation with temperature during the previous and current year’s growing seasons, while in the second warming phase, it demonstrated a strong negative correlation with temperature. In the second warming phase, tree ring width was negatively correlated with temperature and progressively positively correlated with precipitation and SPEI, especially early in the previous year’s growing season and late in the current year’s growing season. Notably, common species like *A. fargesii*, *Larix chinensis*, and *Pinus tabulaeformis* exhibited a consistent pattern aligned with the overall trend (Figure S2).

## 4. Discussion

### 4.1. Trends in Climate and Tree Growth Rates

Climate warming trends were observed in the two phases in the Loess Plateau, accompanied by a decrease in precipitation and SPEI. Previous studies have documented substantial increases in temperature indices in the Loess Plateau during 1961–2010, indicative of the warming trend in the region [22]. This temperature rise aligns with global warming trends and has persisted into the late 20th century [26,27]. Conversely, precipitation levels have shown a declining pattern, consistent with findings from other studies [28,29]. Anthropogenic warming has contributed to shifts in precipitation frequency across different intensity levels in most regions of the Loess Plateau between 1982 and 2015 [30]. MAP in the Loess Plateau has experienced cyclic fluctuations but overall displays a decreasing trend [31]. Moreover, a decreasing trend in spring and autumn precipitation has been detected in the region from 1961–2016 [28]. These long-term warming conditions have led to changes in precipitation patterns and frequencies, ultimately resulting in a relatively pronounced drying trend [24,32]. The shifts in precipitation frequency highlight the intricate interplay of local and global climate drivers, underscoring the complexity of the changes witnessed in the Loess Plateau. Notably, while most regions in Northern China have shown a wetter trend, the Loess Plateau has experienced increased aridity between 1961 and 2017 [33]. Future climate change scenarios suggest that this aridity trend may intensify in the region [34]. This alignment with global trends suggests that the Loess Plateau is not isolated from broader climate dynamics, and its vulnerability to climate change impacts becomes increasingly evident.

In the Loess Plateau, tree growth responded differently to the two warming phases in the 20th century. Specifically, tree growth rates were higher during the first warming phase but declined during the second phase, suggesting contrasting effects of these warming periods on tree growth. Similar findings have been reported in previous studies, such as the diminishing positive effects of spring temperatures on tree growth in the Shennongjia Mountains of China since the late 1970s [35]. *Picea likiangensis* in humid Southwestern China did not show a trend of decline under climate change, whereas spruce trees in water-limited areas would show growth decline [36]. These findings align with our data and indicate that tree growth is influenced by climate warming. Additionally, the effects of warming on tree growth rates vary across different regions and phases in China’s diverse climate zones. For instance, while tree growth in humid Southwestern China did not exhibit a declining trend under changing climates, tree growth in the water-limited Loess Plateau experienced a decline [37]. These studies illustrate the need for area-specific assessments of the impacts of climate change on forest ecosystems, in particular climate change-induced changes in tree growth.

### 4.2. Variations of Tree Growth-Climate Relationships

The trends in tree growth rates during the two warming phases deviate from the consistent climate change trends observed. During the first warming phase, tree growth rates were higher, likely due to the relatively warm and humid climate with a small temperature increase. Additionally, trees did not experience significant drought stress during this phase. These findings suggest that moderate warming can be beneficial for tree growth. This perspective is supported by numerous studies conducted in different regions. For instance, in temperate regions, an increase in standard temperature has been shown to enhance tree photosynthesis rates [37,38]. In the Tibetan Plateau, warmer temperatures have significantly increased gross primary productivity through an increase in leaf area index [39]. It is likely that the initial temperature and aridity level stimulate physiological processes that promote tree growth, such as improving water use efficiency, enhancing light utilization, and increasing leaf area [40,41]. However, an ongoing trend of warming and drying persists during the secondary phase of warming. This extended pattern of warming–drying has the potential to negatively impact tree growth. Excessively high temperatures have been observed to hinder tree growth [42]. Elevated temperatures accelerate water evaporation, leading to soil drought. This phenomenon limits the availability of water and essential nutrients needed to sustain regular growth patterns in trees. Conversely, escalated drought stress also proves to be deleterious to tree growth. This notion is underscored by a study emphasizing that the prolonged elevation in climatic temperatures stands as the primary catalyst for the decline in growth within *Pyrenean silver* fir forests [43]. The escalation in climatic temperatures has the potential to amplify tree mortality rates, particularly in warmer and drier regions [19]. Forests in semi-arid regions are more vulnerable to climate warming, further augmenting the frequency of loss in tree ring formations [44]. Collectively, these discoveries collectively emphasize a sustained trajectory of warming and drying as the chief instigator behind tree growth decline and even mortality.

The impact of climate warming on tree growth varies depending on the phase. In the first warming phase, temperature plays a dominant role, while in the second warming phase, water availability (precipitation and SPEI) becomes a significant factor. Previous studies have shown a notable decline in the correlation between tree growth and temperature in drylands between 1960 and 1990, indicating the presence of other factors limiting tree growth during the later warming phase [45]. However, the response of the tree ring index to precipitation and SPEI becomes more pronounced during this phase, suggesting that water availability becomes the primary limiting factor for tree growth. There are two possible reasons for this shift. Firstly, continued warming leads to increased evapotranspiration and a greater demand for water in the atmosphere [3,30]. This can be attributed to the combined effects of reduced tree growth rates, and accelerated evaporation rates driven by ongoing warming. Secondly, as warming and drying persist, precipitation patterns may undergo changes, resulting in reduced rainfall in drylands [46]. A previous study investigating the effects of temperature and precipitation trends on drought in the Loess Plateau region from 1961 to 2010 revealed that increased temperatures, coupled with limited reduction in precipitation, aggravated drought conditions in the area [22]. Therefore, the severe drought experienced during the later warming phase is the key factor for the shift in tree growth from temperature to water availability (precipitation and SPEI) in the Loess Plateau.

### 4.3. Response of Tree Radial Growth to Climate

Tree response to climate varies across months. The month-to-month climate change for the two warming phases is also shown in Figure S3. In the first warming phase, the temperature signal of tree ring data is most prominent in the current and previous year’s growing season. However, in the second warming phase, the water signal of the tree rings index was mainly concentrated early in the current growing season and late in the previous growing season. In the first warming phase, warming promotes tree growth during the growing season. Therefore, trees are sensitive to temperature throughout the growing season. In the second warming phase, tree growth rates decreased, and tree chronology data were more sensitive to water availability. This implies that tree growth is under drought stress. Several studies have highlighted the importance of growing season precipitation in tree growth under drought conditions. For example, deciduous broadleaf forests in temperate regions primarily experience limited stand productivity due to current growing season water availability [47]. Likewise, in dryland ecosystems of the Silk Road Economic Belt, exacerbated drought enhanced the sensitivity of tree growth to early and mid-growing season precipitation [48]. These studies have demonstrated that with increasing drought severity, growing season precipitation plays an important role in tree growth. Similar findings supporting this idea have been observed in the Loess Plateau region. For instance, the radial growth of *Pinus armandii* in this area is positively correlated with precipitation throughout the growing season [25]. A controlled experiment to investigate the response of tree growth to precipitation found that supplemental water can greatly alleviate drought stress in trees [49]. Our data reveal that tree growth response to drought is weaker during the middle of the growing season compared to the early and late growing seasons. This is likely due to the concentration of precipitation in summer months in the Loess Plateau region, resulting in milder drought stress for trees in the middle of the growing season. Furthermore, an analysis of precipitation patterns in the Loess Plateau from 1965 to 2014 indicates spatial and temporal variability, with precipitation mainly concentrated in the summer months [50].

There is substantial evidence of forest decline due to warm drying, including the widespread decline of *Robinia Pseudoacacia* forests and soil desiccation [51,52,53]. This also suggests that climate warming has irreversible effects on forest ecosystems. It is important to note that our tree ring data are primarily derived from natural forests in the Loess Plateau region. However, it is essential to acknowledge that the Loess Plateau region has been implementing the Grain for Green program since 1999, resulting in large areas of plantation forests. These plantation forests may face substantial challenges in adapting to future climate conditions, necessitating a thoughtful reassessment of forest management strategies [54,55]. With further warming, drought stress in the Loess Plateau region is expected to increase and potentially subject forests to unprecedented drought stress [41,56]. Other water-limited regions are also at risk of facing similar challenges. Therefore, the management of forests to cope with climate change becomes of utmost importance. Certain adaptive forest management practices, such as thinning and land preparation, have already been implemented on the Loess Plateau with favorable outcomes [23,57,58]. Therefore, future forest management ought to not only take into account the effects of continued warming but also adopt scientific and effective management approaches. Furthermore, these findings have profound implications for carbon cycling and storage within ecosystems, ultimately influencing the global carbon balance. The response of forests to climate change in the Loess Plateau and other regions has far-reaching consequences for carbon sequestration and greenhouse gas emissions, making it imperative to address these issues within the broader context of climate mitigation and ecosystem management [43,59,60,61].

## 5. Conclusions

This study demonstrated how climate warming affects tree growth in two warming phases and tested our hypothesis that warming would alter tree growth rates and climate drivers in the Loess Plateau region. It was elucidated that in the two warming phases, temperature exhibited an increasing trend, while precipitation and SPEI demonstrated a decreasing trend. Tree growth rates changed from an increase to a subsequent decreasing trend during the two warming phases in the Loess Plateau. This shift in tree growth sensitivity moved from an initial responsiveness to temperature to a heightened sensitivity to drought stress. Moreover, the first warming phase temperatures promoted tree growth in the previous and current year's growing seasons, while the second warming phase drought stress was concentrated in the early and late growing seasons. Due to the projected intensification of warming, the Loess Plateau region is likely to experience increased aridity, amplifying stress on tree growth and potentially leading to forest decline and mortality. These findings carry practical implications for designing conservation strategies and forest management approaches, as well as for enhancing water resource management in dryland environments.

## Data Availability

The data presented in this study are available on request from the corresponding author. The data are not publicly available yet as the authors are writing some other papers examining these data.

## Supplementary Materials

The following supporting information can be downloaded at: https://www.mdpi.com/article/10.3390/biology12101275/s1, Table S1: Information about the 53 tree-ring chronologies in the Loess Plateau; Table S2: The comparison of tree ring indices, growth rate and climatic sensitivity during the two warming phases in the Loess Plateau; Figure S1: Trends in growth rates of different tree species in two warming phases; Figure S2: Correlation of tree rings index with temperature and precipitation for different tree species in two warming phases; Figure S3: Monthly changes in different climate factors during the two warming phases. All references in the supplementary material are in the text references [62,63,64,65,66,67,68,69,70,71,72,73,74,75,76,77,78,79,80,81,82,83,84,85,86,87,88,89,90,91,92,93,94,95,96].
