# Supplementary material for: Climatic Warming-Induced Drought Stress Has Resulted in the Transition of Tree Growth Sensitivity from Temperature to Precipitation in the Loess Plateau of China
Source: Biology (Basel). 2023 Sep 25;12(10):1275. doi: 10.3390/biology12101275 (PMC10604754; doi:10.3390/biology12101275)
Supplement: Supplementary file 1 [file biology-12-01275-s001.zip › biology-2604121-supplementary.pdf]

Table. S1 Information about the 53 tree-ring chronologies in the Loess Plateau.

| Order | Longitude (°) | Latitude (°) | Precipitation (mm) | Temperature (℃) | Elevation (m) | Tree species | refs cites |
|---|---|---|---|---|---|---|---|
| 1 | 109.83 | 34.30 | | | 2600 | *Abies chensiensis* | Cai *et al.*, 2014 |
| 2 | 108.03 | 33.88 | 1018.1 | 6.4 | 2540 | *Abies fargesii* | Dang *et al.*, 2007 |
| 3 | 108.03 | 33.88 | 1047.5 | 5.2 | 2350 | *Abies fargesii* | Dang *et al.*, 2007 |
| 4 | 108.03 | 33.88 | 1083.2 | 3.7 | 2360 | *Abies fargesii* | Dang *et al.*, 2007 |
| 5 | 108.03 | 33.88 | 1016.8 | 6.5 | 2480 | *Abies fargesii* | Dang *et al.*, 2007 |
| 6 | 108.03 | 33.88 | 1032.6 | 5.9 | 2760 | *Abies fargesii* | Dang *et al.*, 2007 |
| 7 | 108.03 | 33.88 | 1070.5 | 4.3 | 2670 | *Abies fargesii* | Dang *et al.*, 2007 |
| 8 | 109.28 | 40.41 | 256.0 | 7.1 | 1474 | *Juniperus rigida* | Dang *et al.*, 2007 |
| 9 | 107.80 | 34.00 | 850.0 | 1.9 | 3216 | *Larix chinensis* | Qi *et al.*, 2020 |
| 10 | 107.80 | 33.98 | | | 3200 | *Larix chinensis* | Su *et al.*, 2018 |
| 11 | 107.80 | 33.98 | | | 3300 | *Larix chinensis* | Su *et al.*, 2018 |
| 12 | 107.77 | 33.93 | | | 3270 | *Larix chinensis* | Sun *et al.*, 2010 |
| 13 | 107.81 | 34.00 | | | 3200 | *Larix chinensis* | Zhu, 2019 |
| 14 | 107.78 | 33.93 | | | 3200 | *Larix chinensis* | Zhu, 2019 |
| 15 | 111.45 | 37.83 | 463.8 | 9.7 | 2200 | *Larix principisrupprechtii* | Cai *et al.*, 2013a |
| 16 | 111.92 | 38.75 | | | 2621 | *Larix principis-rupprechtii* | Li *et al.*, 2016 |
| 17 | 111.90 | 38.73 | | | 2339 | *Larix principis-rupprechtii* | Li *et al.*, 2016 |
| 18 | 109.51 | 40.40 | 306.5 | 7.1 | 1450 | *Platycladus orientalis* | Sun *et al.*, 2021 |
| 19 | 101.57 | 37.49 | 318.0 | | 2750 | *Picea crassifolia* | Liu *et al.*, 2021 |
| 20 | 101.57 | 37.48 | 318.0 | | 3000 | *Picea crassifolia* | Liu *et al.*, 2021 |
| 21 | 101.91 | 37.70 | 167.0 | 8.0 | 2750 | *Picea crassifolia* | Deng *et al.*, 2013 |
| 22 | 111.45 | 37.83 | 463.8 | 9.7 | 2200 | *Picea meyeri* | Cai *et al.*, 2013b |

| | | | | | | | |
|---|---|---|---|---|---|---|---|
| 23 | 103.40 | 35.24 | | | 2600 | *Picea purpurea* | Ren *et al.*, 2014 |
| 24 | 104.24 | 34.99 | 511.0 | 5.1 | 2350 | *Picea purpurea* | Song *et al.*, 2021 |
| 25 | 104.24 | 34.99 | 510.0 | 5.94 | 2350 | *Picea purpurea* | Sun *et al.*, 2018 |
| 26 | 112.37 | 39.36 | | | 1762 | *Picea wilsonii* | Zhang *et al.*, 2018 |
| 27 | 104.07 | 35.78 | | | 2350 | *Picea wilsonii* | Chen *et al.*, 2015 |
| 28 | 110.08 | 34.47 | | | | *Pinus armandii* | ITRDB |
| 29 | 110.08 | 34.47 | | | | *Pinus armandii* | ITRDB |
| 30 | 112.40 | 36.90 | | | 1450 | *Pinus tabulaeformis* | Cai *et al.*, 2014 |
| 31 | 106.53 | 35.52 | 503.0 | 8.8 | 2000 | *Pinus tabulaeformis* | Song *et al.*, 2011 |
| 32 | 110.07 | 34.47 | 900.0 | 5.9 | 2082 | *Pinus tabulaeformis* | Liu *et al.*, 2009 |
| 33 | 111.33 | 37.77 | 463.8 | 9.7 | 1820 | *Pinus tabulaeformis* | Cai *et al.*, 2013b |
| 34 | 102.43 | 36.41 | | | | *Pinus tabulaeformis* | Wang *et al.*, 2009a |
| 35 | 106.27 | 37.30 | 240.6 | 9.2 | 2400 | *Pinus tabulaeformis* | Wang *et al.*, 2013 |
| 36 | 111.97 | 38.70 | | | 1800 | *Pinus tabulaeformis* | Fang *et al.*, 2009 |
| 37 | 112.08 | 38.83 | | | 1900 | *Pinus tabulaeformis* | Li *et al.*, 2015a |
| 38 | 102.65 | 36.83 | 436.5 | 2.3 | 2200 | *Pinus tabulaeformis* | Li *et al.*, 2012 |
| 39 | 113.45 | 37.40 | 520.0 | 10.0 | 1440 | *Pinus tabulaeformis* | Cai *et al.*, 2013a |
| 40 | 103.80 | 37.40 | 306.7 | 4.9 | 2500 | *Pinus tabulaeformis* | Gao *et al.*, 2005 |
| 41 | 106.27 | 37.30 | 240.6 | 9.2 | 2400 | *Pinus tabulaeformis* | Wang *et al.*, 2013 |
| 42 | 112.08 | 38.83 | | | 1800 | *Pinus tabulaeformis* | Li *et al.*, 2015b |
| 43 | 106.51 | 35.54 | 494.6 | 8.6 | 1800 | *Pinus tabulaeformis* | Fang *et al.*, 2012 |
| 44 | 106.16 | 37.19 | 271.9 | 8.9 | 2300 | *Pinus tabulaeformis* | Wang *et al.*, 2009b |
| 45 | 112.08 | 38.83 | 420.0 | 5.0 | 2400 | *Pinus tabulaeformis* | Li *et al.*, 2006 |
| 46 | 109.78 | 35.65 | 611.8 | 8.6 | 1427 | *Pinus tabulaeformis* | Cai *et al.*, 2005 |
| 47 | 106.27 | 37.28 | 240.6 | 9.2 | 2600 | *Pinus tabulaeformis* | Wang *et al.*, 2013 |

| | | | | | | | |
|---|---|---|---|---|---|---|---|
| 48 | 106.15 | 34.45 | | | 2080 | *Pinus tabulaeformis* | Chen *et al.*, 2014 |
| 49 | 110.42 | 39.29 | 400.0 | 7.0 | 1347 | *Pinus tabulaeformis* | Liang *et al.*, 2004 |
| 50 | 111.72 | 38.97 | | | 1745 | *Pinus tabulaeformis* | Zhang *et al.*, 2018 |
| 51 | 106.10 | 35.58 | 425.0 | 6.3 | 1753 | *Pinus tabulaeformis* | Wang *et al.*, 2009 |
| 52 | 103.39 | 35.23 | | | 2589 | *Sabina przewalskii* | Fang *et al.*, 2014 |
| 53 | 102.31 | 36.59 | | | 3100 | *Sabina przewalskii* | Zhang *et al.*, 2009 |

All references in the supplementary material are in the text references 62-96.

**Table. S2** The comparison of tree ring indices, growth rate and climatic sensitivity during the two warming phases in the Loess Plateau.

| Tree growth rate and climatic sensitivity | 1910 - 1940<br>Mean ± SD | 1970 - 2000<br>Mean ± SD |
|---|---|---|
| Tree-ring indices | -0.010 ± 0.984 | -0.089 ± 0.961 |
| Tree growth rate | 0.027 ± 0.020 | -0.016 ± 0.032 |
| Climatic sensitivity-temperature | 0.082 ± 0.206 | -0.131 ± 0.232 |
| Climatic sensitivity-precipitation | -0.021 ± 0.226 | 0.073 ± 0.225 |
| Climatic sensitivity-SPEI | -0.085 ± 0.224 | 0.077 ± 0.240 |

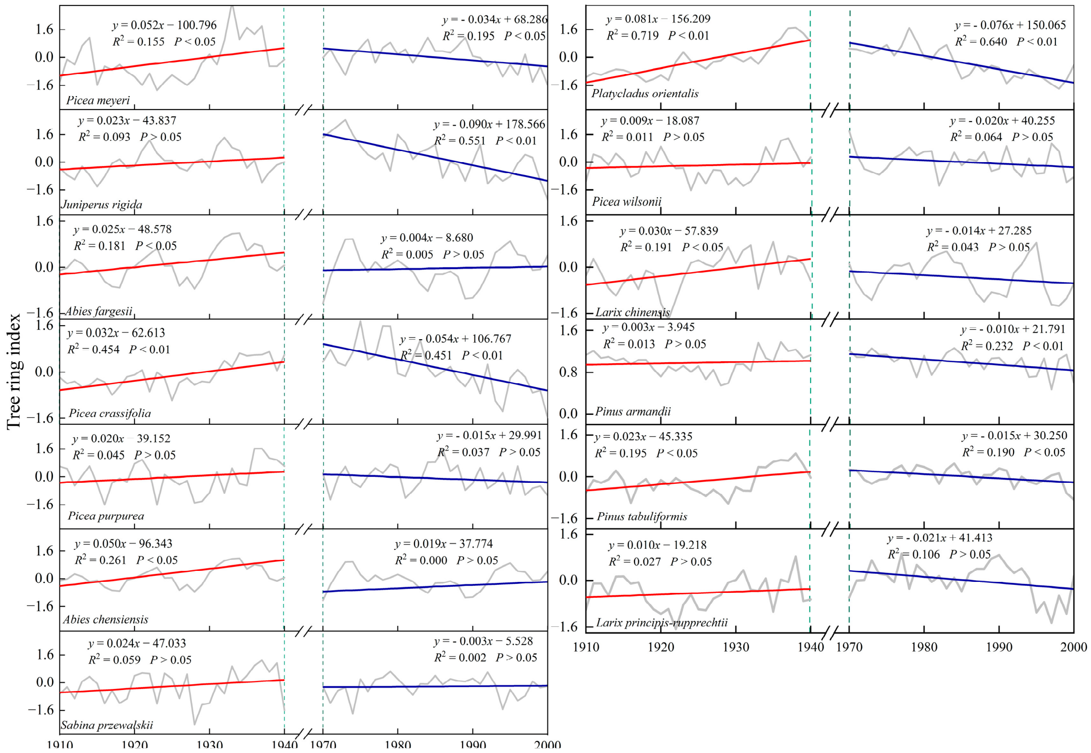

**Figure S1.** Trends in growth rates of different tree species in two warming phases.

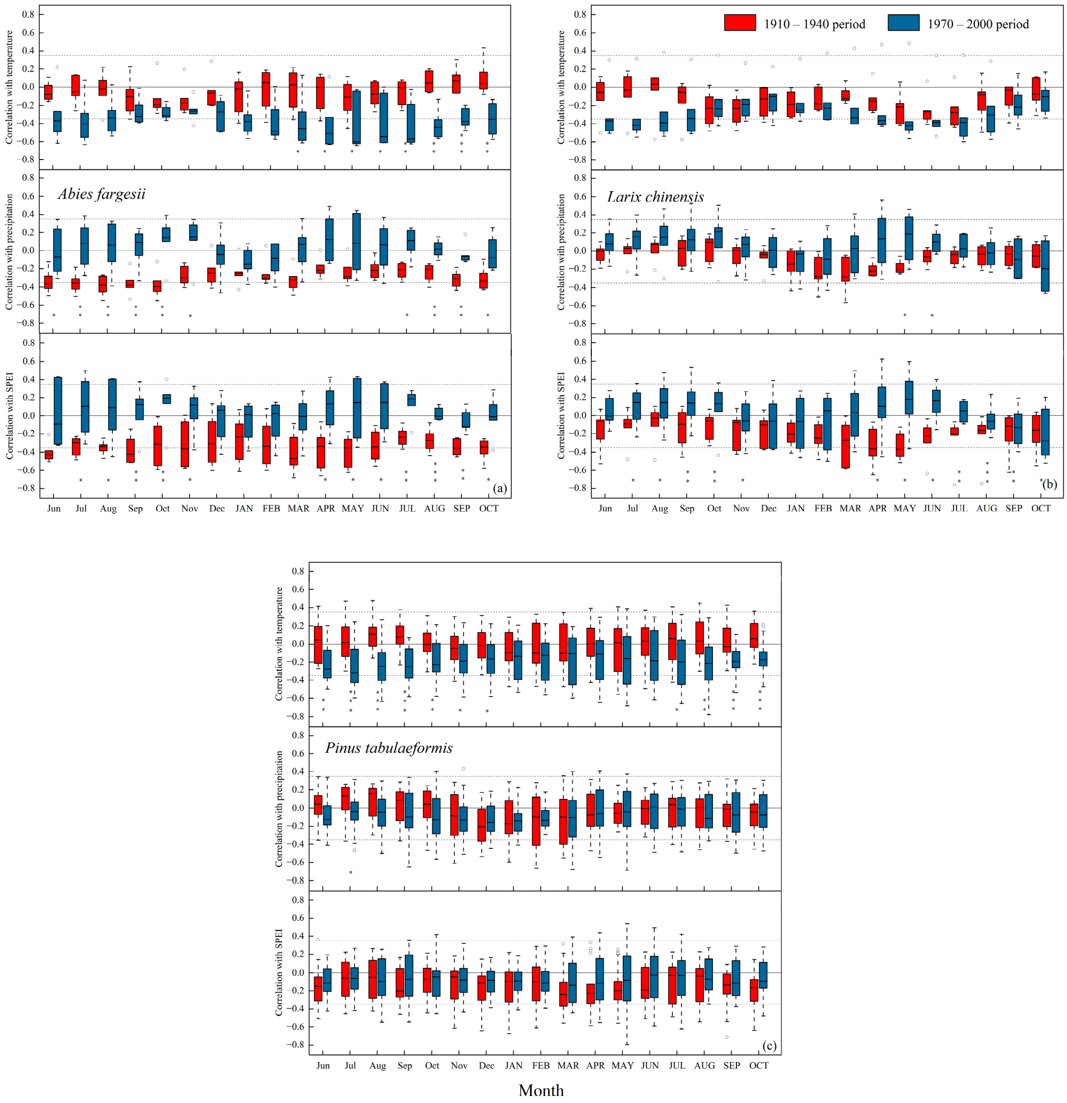


**Figure S2.** Correlation of tree rings index with temperature and precipitation for different tree species in two warming phases.

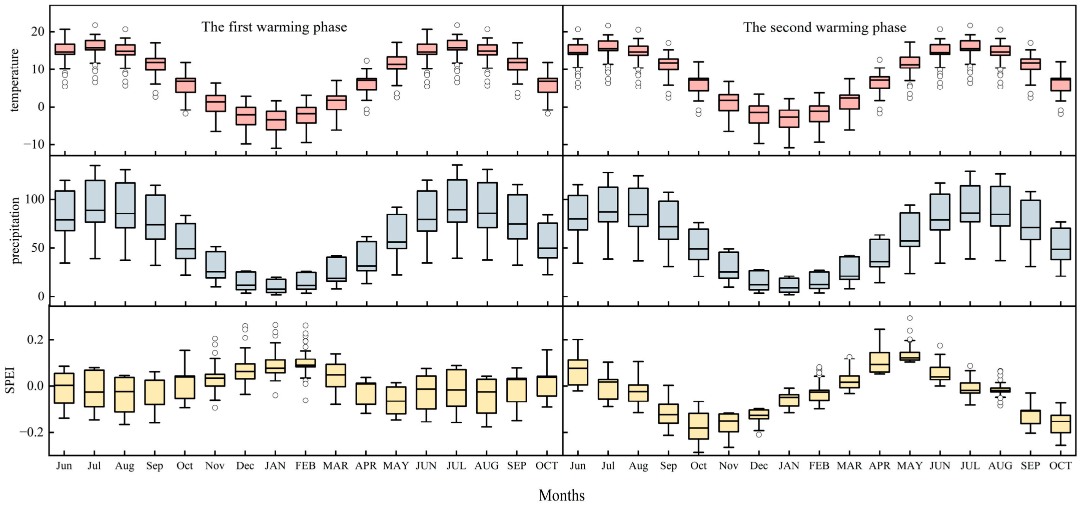


**Figure S3.** Monthly changes in different climate factors during the two warming phases.
